# Shrimp Plasma CREG Is a Hemocyte Activation Factor

**DOI:** 10.3389/fimmu.2021.707770

**Published:** 2021-08-16

**Authors:** Zhiqi Huang, Peng Yang, Fan Wang

**Affiliations:** ^1^Department of Biology, College of Science, Shantou University, Shantou, China; ^2^Institute of Marine Sciences and Guangdong Provincial Key Laboratory of Marine Biotechnology, Shantou University, Shantou, China; ^3^STU-UMT Joint Shellfish Research Laboratory, Shantou University, Shantou, China

**Keywords:** *Penaeus vannamei*, CREG, phagocytosis, transcriptome, hemocyte activation

## Abstract

Cytokines are a class of immunoregulatory proteins that are secreted by cells. Although vertebrate cytokine, especially mammalian cytokine has been well studied for the past decades. Much less attention has been paid to invertebrate so that only some cytokines have been identified in invertebrates. We have chosen *Peaneus vannamei* as a model to explore novel invertebrate cytokines. To achieve this, we previously purified shrimp plasma low abundance proteins and identified more than 400 proteins with proteomics analyses. In this study, a cellular repressor of E1A-stimulated gene (CREG)-like protein, which is highly conserved from *Drosophila melanogaster* to *Homo sapiens*, was further characterized in shrimp plasma. We found that shrimp plasma CREG was a glycoprotein which was strongly induced in hemolymph at 8 h post-LPS injection. Further function experiment unveiled that recombinant shrimp CREG protein injection significantly increased phagocytic hemocyte and lysosome-high hemocyte proportion in hemolymph. After that, hemocytes from rEGFP- and rCREG-protein injected shrimps were subjected to transcriptome analyses, which revealed that shrimp CREG protein could comprehensively promote hemocyte maturation and activation. Taken together, our data clearly indicated that shrimp plasma CREG protein is a novel hemocyte activation factor, which is probably a conserved myeloid cell lineage activation factor from invertebrate to vertebrate.

## Introduction

The cellular repressor of E1A-stimulated genes (CREG) is a multifunctional glycoprotein which is conserved from *Drosophila melanogaster* to *Homo sapiens*. Deletion of this gene could cause embryonic lethality both in *Drosophila melanogaster* (1) and in *Mus musculus* (2), which indicated that this protein played essential roles in development. *In vitro* studies unveiled that the majority of this protein was localized in the endocytic-lysosomal compartment and about 10% was secreted. Overexpression of CREG in human teratocarcinoma NTERA-2 cells decreased cell proliferation by about 60% (3); overexpression of CREG in embryonal carcinoma cells promoted them into the neuronal lineage (4), and it could also induce cardiomyogenic differentiation from embryonic stem cell (5). Taken together, CREG protein could inhibit cell proliferation and induce cell differentiation and senescence in mammals (2). While this protein has shown such various promising functions, a lot of questions about its function remain unsolved at this moment. This protein has, for example, been confirmed as a serum protein in mouse *via* proteomics analysis (6). However, its function as a serum protein remains largely unknown. One possible difficulty is that more than 200 cytokines coexist in mammal serum, which might hinder CREG protein serum functional study.

*Penaeus vannamei*, a major mariculture species in Asia and America, has attracted more and more attention for the past decades due to its increasing culture scale and market demand (7). As an invertebrate animal, its primitive immune system makes it vulnerable to be killed by various environmental pathogens, and frequent diseases have hampered the development of shrimp culture industry, which drives people to explore shrimp immunity (8). Here, we have applied this species as an invertebrate model to explore conserved potential novel cytokines in metazoans. To achieve this, we have previously purified *Penaeus vannamei* low abundant proteins, identified them with mass spectrometry (9), and screened potential functional cytokines with bioinformatics and biochemical methods (10). In this study, a conserved CREG-like protein was identified in shrimp plasma. To our surprise, injection of recombinant shrimp CREG protein could effectively induce hemocyte activation, which clearly indicates that CREG is a conserved pleotropic cell activation factor. It not only plays its role in development stage but also works as an immune cell activation factor and plays its essential roles in innate immunity.

## Materials And Methods

### Experimental Organisms

The shrimps (*Penaeus vannamei*, around 5 g) were purchased from Shantou local farms. Upon delivery, the shrimps were cultured in breeding house tanks filled with aerated seawater at 20°C and acclimatized for 2–3 days before the experiments.

### CREG Sequence Analyses

The coding sequence of shrimp CREG (ROT67215.1) was retrieved from the reported *Penaeus vannamei* genome data (11) and compared with other species *via* the BLAST program (http://www.ncbi.nlm.nih.gov/BLAST/). The simple modular architecture research tool (SMART) online program (http://smart.embl-heidelberg.de) was further used to analyze the protein domain. Multiple sequence alignment was performed using the DNAMAN program (version 6.0.3.99). A neighbor-joining phylogenic tree was constructed based on the amino acid sequences of CREG protein using the MEGA 7.0 software. Bootstrap values were determined based on 1,000 replications. The MS/MS spectrum was retrieved from previous collected plasma proteomics data (9).

### Shrimp Plasma CREG Measurement

Two hundred shrimps were equally divided into two groups and were intramuscularly injected between the second and third segments of the muscle with 100 μl of sterile water or lipopolysaccharide (1 µg/g), respectively (12). Hemolymph samples were collected from 20 shrimps each group at 0, 8, 24, and 48 h postinjection with optimized anticoagulant solution (13) (27 mM citric acid trisodium, 33 mM citric acid, 110 mM d-(+)-glucose, 140 mM sodium chloride, pH 6.0) and immediately centrifuged at 800×*g* for 10 min at 4°C to remove hemocytes. The supernatant plasma was further centrifuged at 550,000×*g* for 2 h with Optima L-100K (Beckman, Brea, CA, USA) to remove most hemocyanin. After that, the clarifying solutions were analyzed with Western blot. To deglycosylate the plasma proteins, 12 μl sample was mixed with 2 μl denaturing buffer (0.5 M DTT, 2.5% SDS) and boiled for 10 min, followed by adding 2 μl sodium phosphate buffer (0.42 M dibasic sodium phosphate, 0.08 M sodium dihydrogen phosphate), 2 μl 10% Triton X-100, and 1 μl peptide-*N*-glycosidase F (0.5 mg/ml, PNGase F) (Yeasen, China). The samples were mixed well and incubated at 37°C for 24 h to remove protein *N*-glycosylation. The deglycosylated samples were analyzed by Western blot.

### SDS-PAGE and Western Blot Analyses

The supernatant samples were separated on SDS-PAGE and transferred to a polyvinylidene fluoride (PVDF) membrane as described before (10). In brief, the membranes were sequentially incubated with rabbit anti-CREG antibody, which is commercially prepared by GenScript, HRP-linked goat anti-rabbit antibody (Sangon Biotech, China), and Western Chemiluminescent HRP substrate (Millipore, Burlington, MA, USA). The signal was detected with an Amersham Imager 600 system (GE Healthcare, North Richland Hills, TX, USA). To detect the total proteins, the PVDF membrane was stained with 0.4% Ponceau S.

### Tissue Distribution of Shrimp CREG Gene

To analyze shrimp (*Peneaus vannamei*, Pv) CREG mRNA abundance in different tissues, hemocytes, hepatopancreas, gill, eyestalk, stomach, heart, intestine, nerve, and muscle were collected and pooled from 15 individual shrimps. Total RNA was purified using a RNAFAST 200 kit (FeiJie, China), and reverse transcribed with a ReverTra Ace qPCR RT Master Mix gDNA Remover kit (Toyobo, Japan) according to the manufacturer’s instructions. The gene-specific primers were designed based on the full-length sequence of PvCREG and PvEF1α (**Table 1**); the tissue distribution of PvCREG was analyzed by quantitative real-time PCR (qPCR) using the Master SYBR Green I system (GenStar, China) on a LightCycler 480 II (Roche, Switzerland). The optimized thermal cycling parameters were 95°C for 10 min, 40 cycles of 95°C for 15 s, and 60°C for 1 min. The qPCR was carried out with three biological replicates with PvEF1α.

**Table 1:** Primers sequences used in this study.

Primer	Sequence (5′-3′)
**qPCR**	
*Pv*CREG-qF	CCAGGCGACTAGTGATCCAT
* Pv*CREG-qR	GAGGCCACAACAACACCTTT
* Pv*PLPP2L-qF	GTGTAGACAGCCATTTGTTTTTC
* Pv*PLPP2L-qR	TGGATGAGAAGACAGATAAGGAT
* Pv*VEGFR2-qF	GGATTCGGAGAGCGTGATAC
* Pv*VEGFR2-qR	CGCTGATGTCTAAAACCACG
* Pv*Src64B2-qF	CTTCTCACAACAGGAGGCTATC
* Pv*Src64B2-qR	TGTGCCGAGGATAAAGTTCA
* Pv*ITGA2B-qF	GGACAATAGCCTGGAACTCG
* Pv*ITGA2B-qR	GCGAGGTATCAGAGGGAACAC
* Pv*SCARBl-qF	CAGAGTCAACCGAAATGCGA
* Pv*SCARBl-qR	CTTGTAGTAGACGGGCACGG
* Pv*ALF-qF	GTAGGCAGAACGACGCAGGA
* Pv*ALF-qR	CGTGTTGTTCTTCTTCGCCTT
* Pv*EFla-qF	TATGCTCCTTTTGGACGTTTTGC
* Pv*EFla-qR	CCTTTTCTGCGGCCTTGGTAG
**dsRNA**	
ds*Pv*CREG-T7F	GGATCCTAATACGACTCACTATAGGCCAGGCGACTAGTGATCCAT
ds*Pv*CREG-T7R	GGATCCTAATACGACTCACTATAGGAACATTGCAGCCTCAGCAAA
ds*Pv*CREG-F	CCAGGCGACTAGTGATCCAT
ds*Pv*CREG-R	AACATTGCAGCCTCAGCAAA
dsEGFP-T7F	GGATCCTAATACGACTCACTATAGGCGTAAACGGCCACAAGTT
dsEGFP-T7R	GGATCCTAATACGACTCACTATAGGTTCACCTTGATGCCGTTC
dsEGFP-F	CGTAAACGGCCACAAGTT
dsEGFP-R	TTCACCTTGATGCCGTTC

### Recombinant Protein Preparation

Shrimp CREG coding sequence was synthesized and cloned into pET-28a by the Beijing Genome Institute (BGI, China). pET-28a-EGFP was from lab stock. Recombinant shrimp CREG and EGFP were sequentially accumulated with Ni-Charged MagBeads (GenScript, Piscataway, CA, USA), washed with PBS containing 1% Triton X-114 for five times, eluted by PBS containing 200 mM imidazole, and dialyzed in PBS to remove the imidazole (14). The recombinant protein endotoxins were assessed before injection with the ToxinSensor™ Chromogenic LAL Endotoxin Assay Kit (GenScript, USA).

### CREG Knockdown Experiment

The RNA interference (RNAi) was performed as previously described with some modifications (10). Briefly, dsCREG and dsEGFP were *in vitro* transcribed using the ScriptMAX^®^ Thermo T7 transcription Kit (Toyobo, Japan) with specific primers (**Table 1**). After that, shrimps were intramuscularly injected with 4 μg/g dsCREG, while the control group shrimps were injected with an equal amount of dsEGFP. At 72 h post-dsRNA injection, the FITC-labeled VP (4 × 10^6^ particles/g VP) was injected into each shrimp. The hemocytes were collected at 8 h postinjection and analyzed with immunoblot or a BD FACSMelody (Becton Dickinson, Franklin Lakes, NJ, USA). For immunoblot, the hemocyte was firstly lysed with lysis buffer (25 mM HEPES, 150 mM NaCl, 1% Triton, 1 mM EDTA, 1 mM PMSF, 1 × protease cocktail (Roche, CHE), pH7.4), and then 12 μl lysate was mixed with 2 μl denaturing buffer (0.5 M DTT, 2.5% SDS) and boiled for 10 min, followed by adding 2 μl sodium phosphate buffer (0.42 M dibasic sodium phosphate, 0.08 M sodium dihydrogen phosphate), 2 μl 10% Triton X-100 and 1 μl peptide-*N*-glycosidase F (0.5 mg/ml, PNGase F) (Yeasen, China). The samples were mixed well and incubated at 37°C for 24 h to remove protein *N*-glycosylation. The deglycosylated samples were analyzed by Western blot. For phagocytic assay, the fluorescence boundary was set based on the detection of the self-fluorescence of untreated hemocytes.

### Lysosome-Stained and Phagocytic Hemocyte Observation

To label lysosome, the hemocytes were collected from 10 shrimps and incubated in staining solution (1 µl Lyso-Tracker Green (Beyotime, China), 133 µl Hoechst 33342 (Solarbio, China), 13.33 ml Insect-XPRESS™ (Lonza, Tampa, FL, USA)) for 1 h. After that, the hemocytes were washed with PBS for two times and observed with a LSM800 confocal microscope (ZEISS, Germany).

To label phagocytic hemocytes, overnight-cultured *Vibrio parahaemolyticus* (VP) was heat killed, labeled with 0.1 mg/ml FITC at 37°C for 1.5 h, washed and resuspended in PBS. The suspension was adjusted to 2 × 10^8^ particles/ml, and 100 μl suspension was injected into each shrimp. The hemocytes were collected from five injected shrimps, stained with Hoechst 33342 at 8 h postinjection and observed with a LSM800 confocal microscope (ZEISS, Germany).

### Lysosome Counts Analyses

The recombinant EGFP and shrimp CREG protein (1 µg/g) were injected into shrimp separately. The hemocytes from each shrimp were collected and stained with Lyso-Tracker Green and Hoechst 33342 at 8 h postinjection, respectively. After that, the hemocytes were analyzed with a BD Accuri™ C6 Plus Flow Cytometer (Becton Dickinson, USA) and quantified with fluorescence intensity. The fluorescence boundary was set based on detection of the self-fluorescence of untreated hemocytes.

### Phagocytic Activity Analyses

The phagocytic assay was developed according to previously described with some modifications (15). In brief, the FITC-labeled VP was mixed with recombinant EGFP and CREG protein separately; 100 µl mixture (1 µg/g protein and 4 × 10^6^ particles/g VP) was injected into each shrimp. The hemocytes were collected at 8 h postinjection and immediately analyzed with a BD Accuri™ C6 Plus Flow Cytometer (Becton Dickinson, USA) and quantified with fluorescence intensity. The fluorescence boundary was set based on detection of the self-fluorescence of untreated hemocytes.

### Transcriptome Sequencing and Analyses

The recombinant EGFP and shrimp CREG protein (1 µg/g) were injected into shrimp, respectively. The hemocytes were collected from 15 shrimps for each treatment at 3 h postinjection and sent to the Beijing Genomics Institute for transcriptome sequencing and analyses. In brief, DNA nanoballs (DNBs) were generated with the single-strand DNA (ssDNA) twined by rolling circle replication (RCR) to enlarge the fluorescent signals at the sequencing process. The DNBs were loaded into the patterned nanoarrays, and pair end reads of 100/150 bp were read through on the BGISEQ platform. For this step, the BGISEQ platform combines the DNA nanoball-based nanoarrays and stepwise sequencing using Combinational Probe Anchor Synthesis Sequencing Method. Assembled sequence data were submitted to GenBank with accession number PRJNA725909. The differentially expressed genes (DEGs) were analyzed as described before with an false-discovery rate (FDR) ≤0.001 and reads per kb per million reads ratio of two samples ≥2 (13).

### Real-Time Quantitative PCR

Real-time quantitative PCR was applied to measure the transcriptional levels of different genes. Each PCR reaction consisted of 1.0 μl cDNA (20 ng/μl), 1.0 μl forward primer (10 μM), 1.0 μl reverse primer (10 μM), 5.0 μl 2× Taq PCR buffer mix, including Taq DNA polymerase, dNTPs and reaction buffer, and 2.0 μl ddH_2_O in 10 μl reaction buffer as recommended by the manufacturer (GenStar, China). The qPCR thermal cycling was performed as previously described. The qPCR was repeated with five biological replicates and analyzed using the 2^−ΔΔCT^ method with the *PvEF1ɑ* gene as the internal control (13). The gene-specific primers are listed in **Table 1**.

### Statistical Analyses

Data are represented as the results of at least three independent experiments. Statistical analyses were performed using GraphPad Prism 8.0. Two-tailed unpaired Student’s *t*-tests were used to calculate significance at *p < 0.05, **p < 0.01, and ***p < 0.001.

## Results

### Identification of Shrimp CREG Protein as an LPS-Induced Plasma Glycoprotein

**Figure 1:**
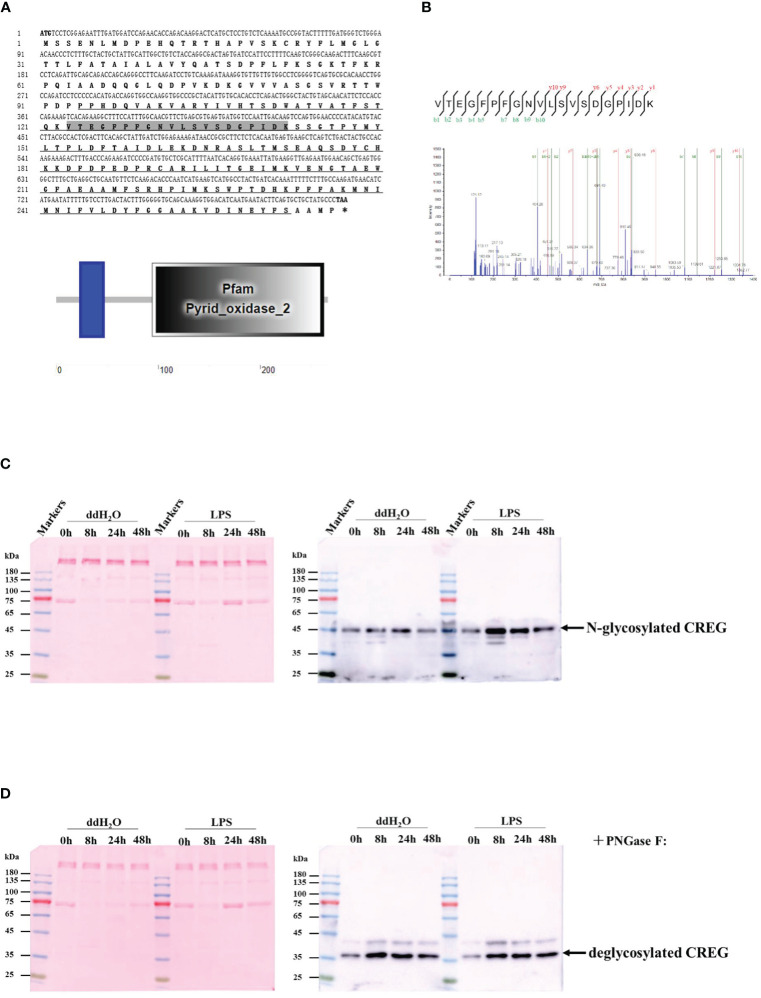
Identification of shrimp cellular repressor of E1A-stimulated gene (CREG) protein as a lipopolysaccharide (LPS)-induced plasma glycoprotein. **(A)** The ORF of the amino acid sequence is shown with one-letter codes. The initiation codon (ATG) and the stop codon (TAA) are shaded. The structural domain prediction of shrimp CREG. **(B)** A peptide of CREG is identified with MS/MS spectrum. Shrimp plasma was collected at 0, 8, 24, and 48 h post-LPS injection, followed by ultracentrifugation to remove hemocyanin. After that, the supernatant was directly loaded for WB detection **(C)** before or **(D)** after deglycosylation. Right, the membrane was blotted with shrimp CREG-specific antibody. Left, the membrane was stained with ponceau as a loading control.

**Figure 2:**
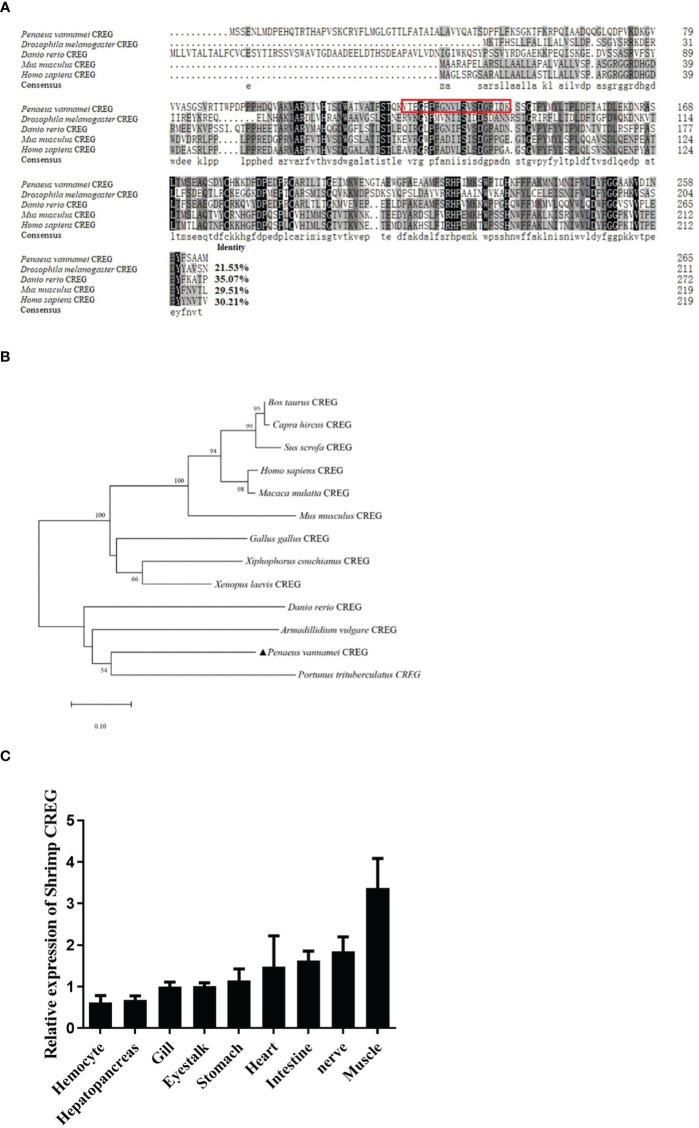
Sequence analyses and tissue distribution of shrimp CREG protein. **(A)** Multiple sequence alignment between shrimp CREG protein and other CREG proteins. CREG proteins used for the analysis include: *Drosophila melanogaster* (NP_001247152.1), *Danio rerio* (NP_001007306.1), *Mus musculus* (NP_035934.1), and *Homo sapiens* (NP_003842.1). Identical amino acid residues are shaded in black and similar residues in gray. **(B)** The neighbor-joining phylogenetic tree based on the sequences of shrimp CREG protein and other known vertebrate and invertebrate CREG proteins. The numbers marked on the tree branches represent the bootstrap values. The location of shrimp CREG protein is indicated by a black-filled triangle. **(C)** Tissue distribution of shrimp CREG protein in shrimps. The expression of shrimp CREG protein was determined relative to PvEF1α expression, as determined using real-time qPCR analysis.

As previously described, we identified a peptide with MS/MS spectrum on shrimp CREG protein, which drove us to retrieve CREG sequence from NCBI (ROT67215.1) (**Figures 1A, B** and **Figure 2A** (red circled)). To confirm whether this protein is a shrimp plasma protein, CREG-specific antibody was raised for plasma CREG protein detection. As shown in **Figure 1C**, CREG protein was significantly accumulated in shrimp plasma at 8 h after lipopolysaccharide (LPS) injection and slowly restored at 24 and 48 h after LPS injection. Double-distilled water (ddH_2_O) injection is a weak stimulus compared with LPS, thus ddH_2_O injection also induced a mild accumulation of shrimp CREG protein in plasma at 8 h postinjection. Theoretical molecular weight of this shrimp CREG protein was 29.5 kDa while Western blot showed that it was a 45-kDa protein. One possible explanation was that this shrimp CREG protein is a glycoprotein, which is similar with its mammalian homolog (3). To justify this, shrimp plasma was treated with peptide *N*-glycosidase F (PNGase F) to remove N-linked glycans from proteins and analyzed by Western blot. As shown in **Figure 1D**, treatment with PNGase F abolished the 45-kDa CREG protein band. Instead, deglycosylated forms of CREG protein were shown as the 35-kDa band and around 42 kDa band. Moreover, these forms of CREG protein were also accumulated at 8 h postinjection, which were consistent with glycosylated CREG protein Western blot. These data indicated that shrimp CREG protein was a LPS-induced plasma glycoprotein.

### Sequence Analyses and Tissue Distribution of Shrimp CREG gene

Since *Penaeus vannamei* is not a model species, multiple sequence alignment was performed to evaluate how conserved this protein was. As shown in **Figure 2A**, *Penaeus vannamei* CREG is similar with *Drosophila melanogaster* (21.53% identity), *Danio rerio* (35.07%), *Mus musculus* (29.51%), and *Homo sapiens* (30.21%) separately. More interestingly, *Penaeus vannamei* CREG shared highest identity with *Danio rerio* (35.07%) instead of *Drosophila melanogaster* (21.53%), and both *Danio rerio* and *Penaeus vannamei* CREG had an extra N-terminal dozens of amino acids compared with other terrestrial animal, which suggest that N-terminal of CREG from *Penaeus vannamei* and *Danio rerio* might play certain roles in aquatic animal development. Further phylogenetic tree analyses indicated that *Penaeus vannamei* CREG has a shortest evolutionary distance with *Portunus trituerculatus* and has a longest evolutionary distance with mammals (**Figure 2B**). In addition, shrimp CREG mRNA relative abundances in different tissues were analyzed by qPCR. As shown in **Figure 2C**, this gene had a moderate expression in all tested tissues. The muscle had the highest expression level of this gene, which was around 4.5 times higher than the lowest level in hemocyte. For other tissues, expression of this gene in nerve, intestine, heart, stomach, eyestalk, gill, and hepatopancreas was 2, 1.6, 1.4, 1.9, 0.6, 0.6, and 0.1 times higher than that in hemocyte. These data indicated that this gene was ubiquitously expressed in shrimp.

### Hemocyte Activation Activity of Recombinant Shrimp CREG Protein

**Figure 3:**
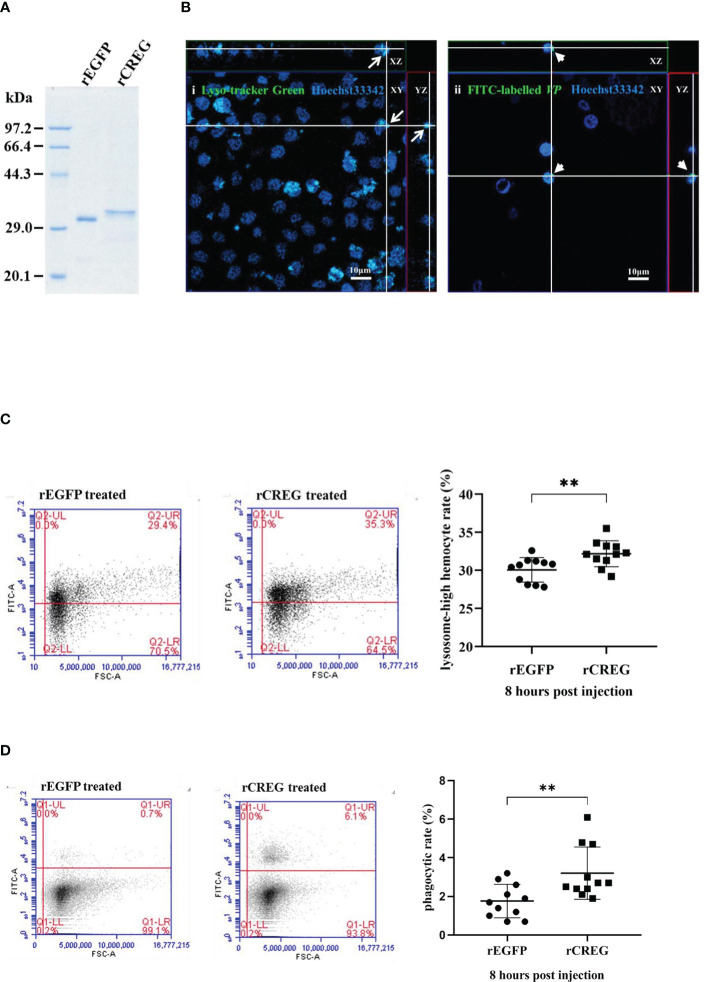
Recombinant shrimp CREG protein induces hemocyte differentiation. **(A)** SDS-PAGE of purified rEGFP and rCREG. **(B)** Confocal microscopy observation of lysosome-labeled (green) (i) and FITC-VP-phagocytized (green) (ii) hemocytes with Hoechst 33342 (blue)-stained nuclei. Labeled lysosomes are indicated by arrows, and phagocytized FITC-VPs are indicated by arrow heads. **(C)** Lysosome^high^ hemocyte proportion of total hemocytes extracted from rEGFP- (control) and rCREG-treated shrimp. Left, the representative scatter plot for the rEGFP-treated shrimp. Middle, the representative scatter plot for the rCREG-treated shrimp. Right, each dot represents the test result of one shrimp. **(D)** The phagocytic activity of total hemocytes extracted from rEGFP + FITC VP-treated shrimp and rCREG + FITC VP-treated shrimp. Left, the representative scatter plot for rEGFP + FITC VP-treated shrimp. Middle, the representative scatter plot for the rCREG + FITC VP-treated shrimp. Right, each dot represents the test result of one shrimp. Each dot represents the test result of one shrimp, **p < 0.01 was calculated by two-tailed unpaired Student’s *t*-tests compared with the control.

As a conserved secreted glycoprotein in shrimp plasma, we wanted to know what roles this protein played in shrimp hemolymph. Because CREG could inhibit cell proliferation and induce cell differentiation in mammals (2), we wanted to know whether this LPS-induced plasma protein played similar roles in shrimp hemolymph. To answer this question, we first purified recombinant shrimp CREG protein and recombinant EGFP protein and analyzed them with SDS-PAGE, which showed that the purities of these two proteins were more than 90% (**Figure 3A**). Next, we checked the literatures and tried to develop some reliable methods to monitor shrimp hemocyte activation because there are no well-recognized lineage markers for shrimp hemocytes until now. More recently, single cell sequencing of mosquito hemocytes unveiled that invertebrate hemocytes could be divided into three major categories: prohemocyte, granulocyte, and oenocytoid, which were mainly characterized with proliferation, phagocytosis, and phenoloxidase activity, respectively (16). In crustacean field, hyaline cell, which was recognized as prohemocyte, and granulocyte/semi-granulocyte, which were regarded as terminal differentiated effector cells, were well-recognized (17). For this prohemocyte/granulocyte/semigranulocyte classification standard, lysosome was believed to be an important marker between prohemocyte and effector cell, because it played critical roles in pathogen elimination (17). Thus, we applied a lysosome marker to evaluate prohemocyte/differentiated hemocyte ratio with Fluorescence Activating Cell Sorter (FACS) assay. Another well-recognized marker was phagocytic activity, which people normally believed that phagocytic cells belonged to differentiated cells and progenitor cells didn’t have phagocytic activity (18). Therefore, we labeled *Vibrio parahemolyticus* (VP), one of the most common shrimp pathogens with fluorescein isothiocyanate (FITC), injected it into shrimp, collected shrimp hemocytes at 8 h postinjection and analyzed them with a fluorescence microscopy and FACS (19). By these two ways, we could evaluate shrimp CREG activation activity *in vivo.* As shown in **Figure 3B**, lysosomes in hemocytes and phagocytic hemocytes were labelled with Lyso-Tracker Green and FITC-VP, respectively. In this way, we could evaluate shrimp hemocyte activation with lysosome quantities and phagocytic activities. Injection of recombinant shrimp CREG protein could effectively increase the proportion of lysosome^high^ hemocytes and phagocytosis^strong^ hemocytes in shrimp hemolymph (**Figures 3C, D**), which clearly indicated that shrimp plasma CREG protein had a hemocyte activation activity.

To further explore CREG function in shrimp, RNA interference experiments were performed. As shown in **Figure S1A**, hemocyte CREG was successfully reduced with dsCREG injection. However, we could not detect a significant reduction for plasma CREG (data not shown). One possible explanation is that the plasma CREG comes from multiple organs (**Figure 2C**). It will be difficult to effectively deliver dsRNA to some tissues. Further phagocytic assay showed that hemocyte CREG knockdown did not affect its phagocytic activity significantly (**Figure S1B**). This might be due to the limited knockdown efficiency and plasma CREG compensation.

### Transcriptome Analyses of Shrimp CREG Protein-Treated Hemocytes

**Figure 4:**
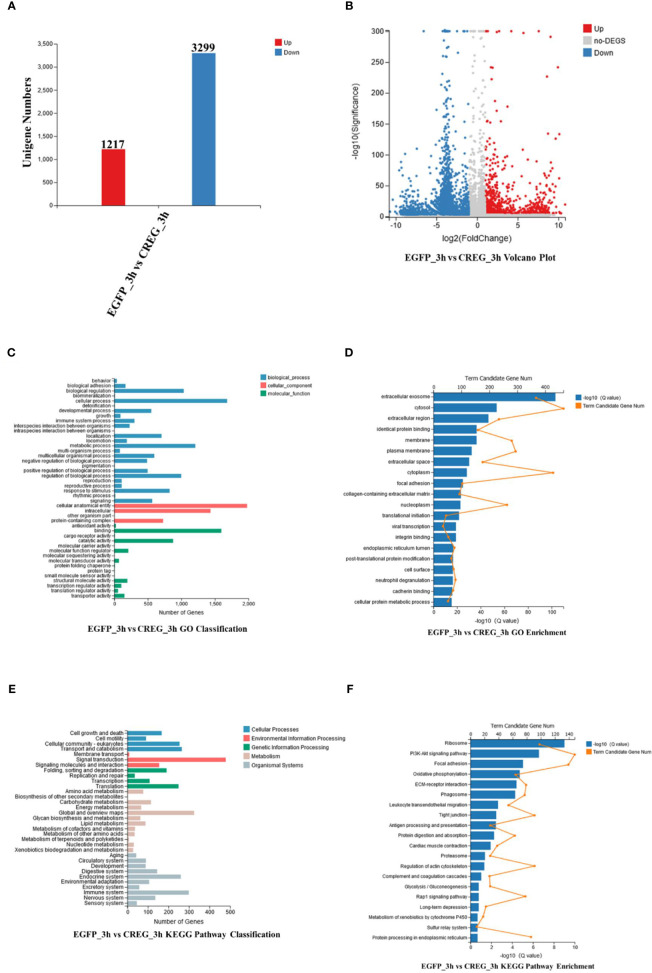
Functional analyses of differentially expressed genes (DEGs) from transcriptomic data. **(A)** Statistical histogram, **(B)** volcano plot, **(C)** GO classification, **(D)** GO enrichment, **(E)** KEGG pathway classification, and **(F)** KEGG pathway enrichment of DEGs. Differentially expressed genes based on adjusted false-discovery rate (FDR) ≤0.001 and log 2FC ≥1.

To further explore what function of this protein, recombinant EGFP and recombinant CREG were injected into shrimps, after that, the hemocytes were collected at 3 h postinjection and subject for transcriptomics analyses. A cutoff of FDR ≤0.001 and log2 FC≥1 was used as the threshold for DEGs(differential expressed genes) selection. As shown in **Figures 4A, B**, totally 1217 upregulated genes and 3299 downregulated genes were screened comparing CREG treatment with EGFP treatment. To further analyze these DEGs, they were annotated with GO and KEGG databases. In GO classification analysis, 1681 DEGs were enriched in cellular process, 1,980 DEGs were enriched in cellular anatomical entity, and 1,596 DEGs were enriched in binding, which suggest that CREG treatment induced intracellular granulation (**Figure 4C**). Further GO enrichment showed that a large number of secreted proteins were changed which included extracellular exosome (367), extracellular region (234), extracellular space (176), focal adhesion (103), collagen-containing extracellular matrix (93), neutrophil degranulation (79). These data unveiled that CREG treatment induced hemocyte maturation and activation (**Figure 4D**). Furthermore, KEGG pathway classification indicated that 479 DEGs were enriched in signal transduction, 325 DEGs were enriched in Global metabolism and 299 DEGs were enriched in immune system. These suggest that CREG treatment has a profound general effect on hemocyte metabolism and activity (**Figure 4E**). In KEGG pathway enrichment analysis, the pathways involved in hemocyte activation were significantly enriched, such as ribosome (98), PI3K-Akt (148), Focal adhesion (139), Oxidative phosphorylation (64), ECM-receptor interaction (79) and phagosome (77) (**Figure 4F**). In general, our transcriptome analyses unraveled that CREG treatment profoundly activated shrimp hemocytes.

### Validation of Differentiation Expressed Genes by qPCR

**Figure 5:**
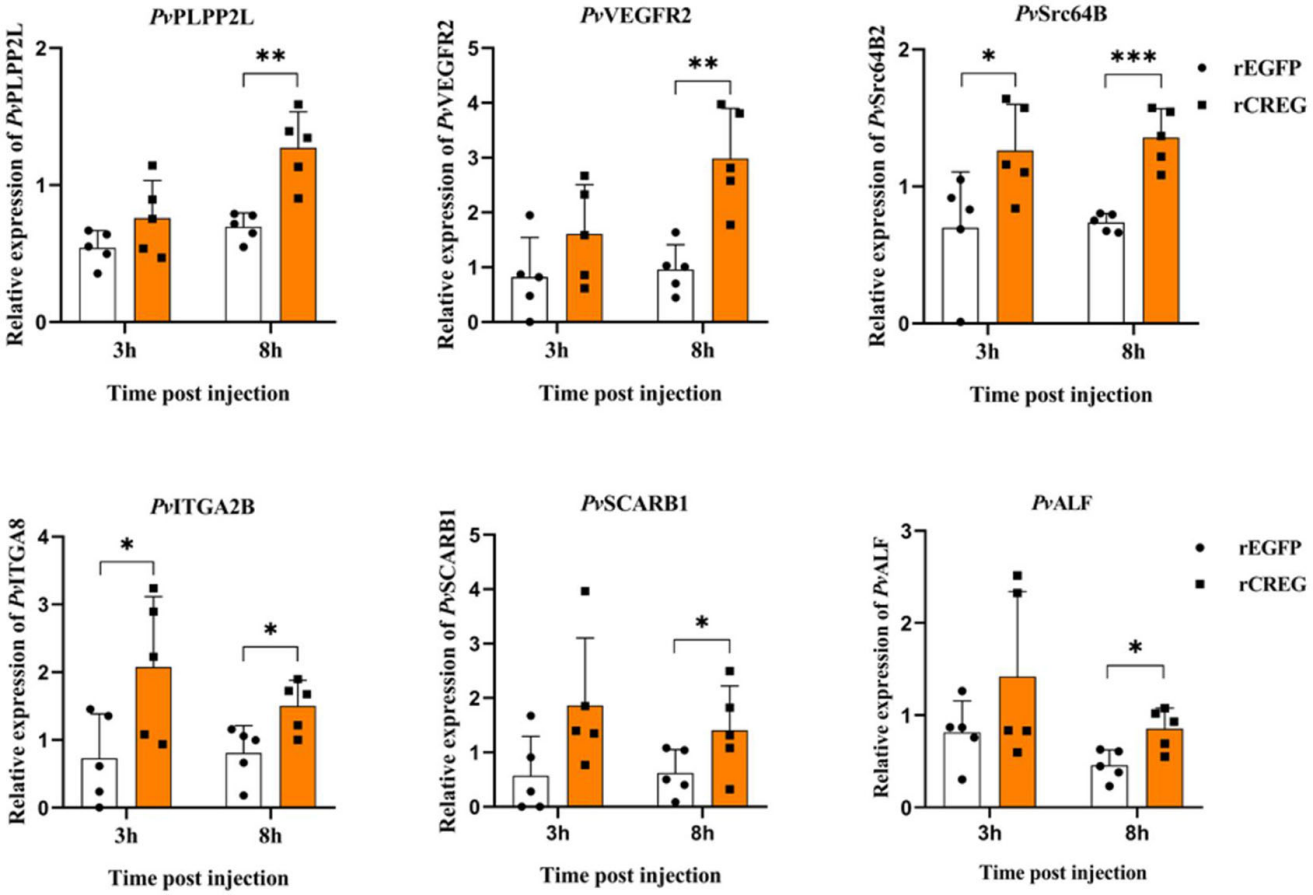
Validation of differentially expressed genes (DEGs) by qPCR. To validate the transcriptome data, the relative gene expression levels of six selected DEGs were examined by qPCR. Each dot represents the test result of one experiment. *p < 0.05, **p < 0.01, and ***p < 0.001 were calculated by two-tailed unpaired Student’s *t*-tests compared with the control (0 h, set as 1.0).

To testify our conclusion, the DEGs which could be potential differentiation markers were chosen for further validation. Phospholipid phosphatase 2-like protein (LOC113830003) (PLPP2L), which regulates the dephosphorylation of lipid phosphates and cell cycle (20), has no significant induction at 3 h postinjection (hpi) and a twofold induction at 8 hpi. Vascular endothelial growth factor receptor 2 (LOC113800792) (VEGFR2), a well-characterized receptor for promoting cell activities (21), has no significant induction at 3 hpi and a threefold induction at 8 hpi. Tyrosine-protein kinase Src64B-like (MH397364.1) (Src64B), a well-characterized oncogene (22), has a two-fold induction both at 3 and 8 hpi. Integrin alpha-IIb-like (LOC113815644) (ITAG2B), a cell surface receptor whose different subtypes have been widely applied as cell lineage markers (23, 24), has a threefold induction at 3 hpi and a twofold induction at 8 hpi. Scavenger receptor class B member 1-like (LOC113802127) (SCARB1), a macrophage surface receptor for lipoprotein (25), has a 2.4-fold induction at 8 hpi. Antilipopolysaccharide factor-like (ROT72950.1) (ALF), an antimicrobial peptide, has a two-fold induction at 8 hpi. In general, our results indicated that recombinant CREG protein could activate shrimp hemocytes and promote their phagocytic activity (**Figure 5**).

## Discussion

In mammals, immune cells could be divided into more than 10 different lineages, and it is mediated by dozens of various cytokines (26). However, invertebrate, which is lack of adaptive immunity, has only three well-recognized hemocyte subtypes, for example, there are three main hemocyte classes: plasmatocytes, crystal cells, and lamellocytes in the circulation of fly larva (27). While in mosquito prohemocytes, oenocytoids, and granulocytes could be distinguished based on morphology (28). In crustacean, hyaline cell, semi-granular cell and granular cell were described (17). More recently, single cell RNA sequencing revealed more subtypes in invertebrate hemocyte typing (16, 29), which raised a question how these subtypes were *in vivo* regulated? In mammals, certain blood cell subtype was frequently regulated by some differentiation factors. However, much less invertebrate regulation factors have been discovered (16), which hindered people’s understanding of invertebrate cellular immunity. Here, we identified a conserved secret glycoprotein in shrimp plasma, which does not only play its differentiation roles in mammal embryonic stem cells (5) but also plays its activation roles in shrimp hemocytes. More recently, people have identified that CREG could promote lysosomal biogenesis and played important roles in both macropinocytosis and clathrin-dependent endocytosis (30), which is consistent with our findings. More interestingly, this protein could be strongly induced in shrimp plasma by LPS and sequentially mediated shrimp hemocyte activation. Our result showed that this CREG protein in shrimp plasma worked like a Macrophage Colony-Stimulating Factor (M-CSF) in mammal, which could effectively stimulate phagocytotic immune cells upon infection. Thus, our data provide a novel invertebrate activation factor that could help people to further understand how invertebrate hemocytes were regulated.

CREG was originally reported as a cellular protein that antagonized transcriptional activation and cellular transformation by the adenovirus E1A protein (31). Later, it was confirmed as a secreted *N*-glycoprotein *(* 32), and extracellular CREG could mediate cell proliferation and differentiation *via* mannose 6-phosphate/insulin-like growth factor II receptor (M6P/IGF2-R) (2). Here, we found that recombinant shrimp CREG without glycosylation could effectively induce hemocyte activation, which suggest that the glycosylation of this protein may not be essential for its activation function. Instead, the glycosylation of shrimp CREG could probably protect this protein from plasma protease degradation and extend its half-life in plasma (33). Moreover, tissue distribution indicated that this protein was expressed in all examined tissues, which suggest that plasma CREG probably came from multiple organs. We do not even make sure that shrimp plasma CREG only activate hemocytes or activate multiple organs *in vivo* at this moment. In addition, shrimp plasma CREG could be strongly induced with LPS injection which implied that this glycosylation could possibly help CREG interact with pathogen-associated molecular patterns (PAMPs) and shrimp CREG might partially work as a pattern recognition molecule (PRM). All these questions remain to be further studied (34).

Invertebrate cellular immunity has been much less studied compared with vertebrate for the past decades. There are many obstacles and technical hurdles in this field. Limited cytokines, for example, have been identified for invertebrate, which mainly came from fly and mosquito. For instance, PDGF- and VEGF-receptor-related ligand (Pvf1) and adenosine-dependent growth factor (Adgf-A) promote prohemocyte differentiation into intermediate hemocytes in fly (27). A putative hemocyte differentiation factor (HDF) has been proposed, whose release in mosquitoes results in a permanent increase in the proportion of circulating granulocytes in hemolymph (35), but until now it has not been fully characterized and may contain Lipoxin A4 (36). Here, we identified another hemocyte activation factor in shrimp, which could be identified from fly to human. Thus, our data shed light on the conserved myeloid lineage regulation studies from invertebrate to vertebrate. CREG would be a nice key, which when combined with recent prevailing single-cell sequencing technology, could unveil common mechanisms for myeloid lineage regulation.

## Data Availability Statement

The datasets presented in this study can be found in online repositories. The names of the repository/repositories and accession number(s) can be found below: https://www.ncbi.nlm.nih.gov/, PRJNA725909.

## Author Contributions

FW: conceptualization, methodology, project administration, supervision, funding acquisition, writing (original draft), and writing (review and editing). ZH: investigation, formal analysis, writing (original draft), and writing (review and editing). ZH performed the experiments and analyzed data with help from PY. All authors contributed to the article and approved the submitted version.

## Funding

This work was sponsored by the National Natural Science Foundation of China (No. 41976123) and the “Sail Plan” Program for the Introduction of Outstanding Talents of Guangdong Province of China (No. 14600703).

## Conflict of Interest

The authors declare that the research was conducted in the absence of any commercial or financial relationships that could be construed as a potential conflict of interest.

## Publisher’s Note

All claims expressed in this article are solely those of the authors and do not necessarily represent those of their affiliated organizations, or those of the publisher, the editors and the reviewers. Any product that may be evaluated in this article, or claim that may be made by its manufacturer, is not guaranteed or endorsed by the publisher.

## Supplementary Material

Supplementary Figure 1 Shrimp hemocyte CREG knockdown do not affect its phagocytic activity significantly. The dsCREG and dsEGFP were injected into shrimps separately. FITC-labeled VP was injected at 72 h post-dsRNA injection. After another 8 h, the hemocytes were collected for immunoblot **(A)** and phagocytic assay **(B)**. Each dot represents the test result of one shrimp. The data were analyzed by two-tailed unpaired Student’s *t*-tests compared with the control.

Supplementary Table 1 Differentially expressed genes between rEGFP- and rCREG-treated shrimp hemocytes.
