# Supplementary figures and images for: Shrimp Plasma CREG Is a Hemocyte Activation Factor
Source: Front Immunol. 2021 Aug 16;12:707770. doi: 10.3389/fimmu.2021.707770 (PMC8415475; doi:10.3389/fimmu.2021.707770)

A.

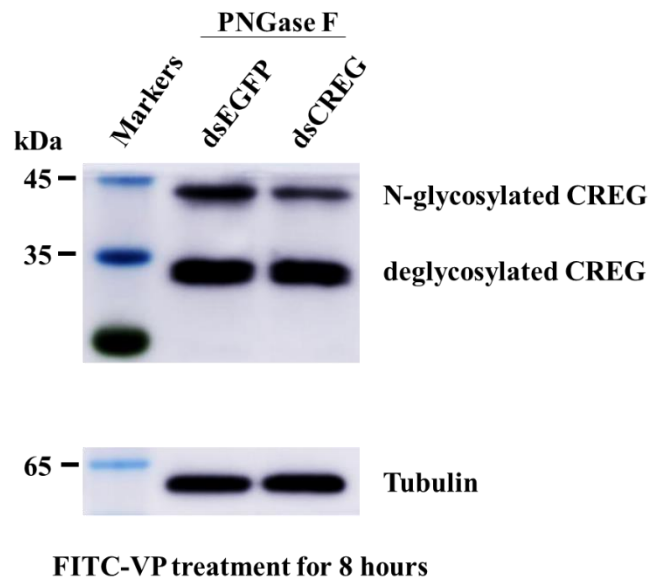

B.

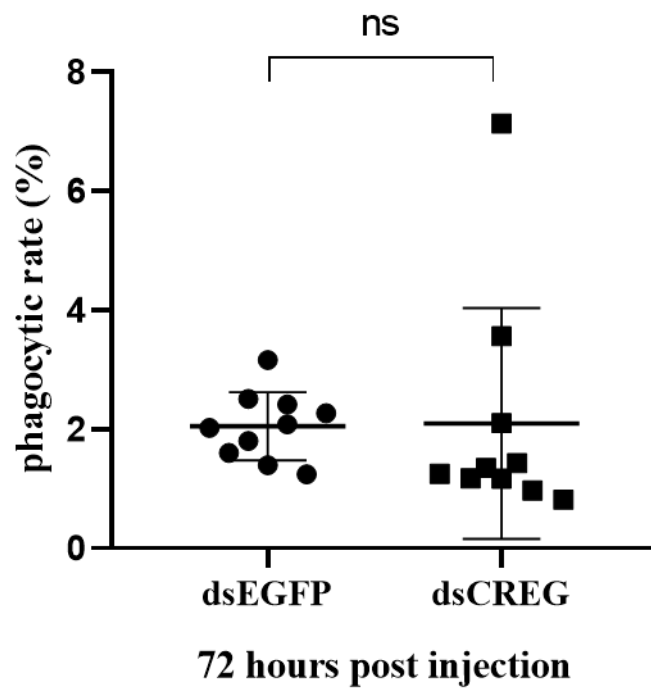

Supplement: Supplementary Figure 1 — Shrimp hemocyte CREG knockdown do not affect its phagocytic activity significantly. The dsCREG and dsEGFP were injected into shrimps separately. FITC-labeled VP was injected at 72 h post-dsRNA injection. After another 8 h, the hemocytes were collected for immunoblot (A) and phagocytic assay (B). Each dot represents the test result of one shrimp. The data were analyzed by two-tailed unpaired Student’s t-tests compared with the control. [file DataSheet_2.pdf]
